# Enhancing safety monitoring in post-stroke rehabilitation through wearable technologies

**DOI:** 10.1177/02692155241309083

**Published:** 2025-01-07

**Authors:** Kátia Rech, Maira Jaqueline da Cunha, Ana Paula Salazar, Rosicler da Rosa Almeida, Clarissa Pedrini Schuch, Gustavo Balbinot

**Affiliations:** 1 Movement Analysis and Rehabilitation Laboratory, 117303 Universidade Federal de Ciências da Saude de Porto Alegre (UFCSPA), Porto Alegre, Brazil; 2 Rehabilitation Sciences Graduate Program, 117303 Universidade Federal de Ciências da Saude de Porto Alegre (UFCSPA), Porto Alegre, Brazil; 3 MyantX Corp, Mississauga, ON, Canada; 4 Department of Biomedical Physiology and Kinesiology, 1763 Simon Fraser University, Burnaby, BC, Canada; 5 Movement Neurorehabilitation and Neurorepair laboratory, 1763 Simon Fraser University, Burnaby, BC, Canada; 6 Institute for Neuroscience and Neurotechnology, Simon Fraser University, Burnaby, BC, Canada

**Keywords:** Mobility, smoothness, stroke, inertial measurement unit, automated classification

## Abstract

**Objective:**

Current clinical practice guidelines support structured, progressive protocols for improving walking after stroke. Technology enables monitoring of exercise and therapy intensity, but safety concerns could also be addressed. This study explores functional mobility in post-stroke individuals using wearable technology to quantify movement smoothness—an indicator of safe mobility.

**Design:**

Observational cohort study.

**Setting:**

A movement analysis and rehabilitation laboratory.

**Participants:**

A total of 56 chronic post-stroke individuals and 51 healthy controls.

**Intervention:**

Participants performed the mobility test while wearing an inertial measurement unit attached to their waist. Thirty-two healthy participants also engaged in a steady-state walking task.

**Main measures:**

Functional mobility smoothness by examining angular velocities in the yaw, pitch, and roll axes, employing the spectral arc length metrics.

**Results:**

Our findings reveal that post-stroke individuals extend the duration of the timed-up-and-go test (≈9 s and 23 s longer compared to the controls) to ensure safe mobility—greater mobility smoothness (*p* < 0.001). Notably, for mild and severe impairments, post-stroke mobility demonstrated ≈8% and ≈11% greater smoothness in pitch movements, respectively (*p* = 0.025 and *p* = 0.002). In the roll direction, mobility was ≈12% smoother in cases of severe strokes (*p* = 0.006).

**Conclusion:**

This study addresses a crucial gap in the understanding of mobility smoothness in chronic stroke survivors using wearable technology. Our study suggests the potential utility of spectral arc length to predict challenging mobility situations in real-world situations. We highlight the potential for automated monitoring of safety offering promising avenues for real-time, real-life monitoring.

## Introduction

Stroke is a leading cause of long-term disability worldwide, with impairments significantly impacting mobility and quality of life.^1^ Assessing these impairments is crucial for guiding rehabilitation strategies and predicting the risk of adverse events such as falls.^2^ The timed-up-and-go test, a widely used clinical assessment, incorporates essential components such as sharp turns and sit-to-stand and stand-to-sit transitions, making it a multifaceted evaluation tool for assessing functional mobility and post-stroke impairments.^3,4^ The most commonly used metric is the time to complete the timed-up-and-go test; a cutoff of 13.49 s effectively differentiates performance from older adults.^5^ The time to complete the task is correlated with Fugl-Meyer and balance scores, as well as with strength in the hip abductor, knee flexor, ankle dorsiflexor, and plantar flexor on the paretic side.^5^

The time required to complete the timed-up-and-go test is the usual parameter used to compute functional mobility performance. More recently, the use of inertial measurement units (IMUs) has allowed for the assessment of other parameters related to motor performance and movement quality (reviewed in Ortega-Bastidas et al.^6^), such as movement smoothness.^7–9^ Poor balance and a higher fall risk have been linked to the absence of smoothness in movements across different groups, such as stroke survivors.^8^ Understanding mobility smoothness is pivotal because it provides insights into movement quality and has the potential to detect risk of falls.^10–12^

Here, we employed the spectral arc length analysis, which provides a quantitative measure of movement smoothness.^7,8,10,13^ We first aimed to explore the relationship between smoothness during steady-state walking and the timed-up-and-go test. We hypothesized that changes in direction, turns, and body weight support changes would lead to reduced smoothness in healthy participants. Next, we quantified smoothness in post-stroke individuals to explore how these participants deal with challenging mobility situations. We hypothesized that individuals with stroke would be more cautious while performing the mobility task, given the lower extremity impairment in the paretic side.^5^

Our previous studies demonstrated the ability of mobility smoothness metrics to predict fall history in the oldest-old individuals,^7,10^ emphasizing their potential to detect fall risks in real time in the future. The capacity to identify fall risks is an important addition to metrics commonly extracted from wearable devices—such as heart rate and distance traveled—and holds significant implications for advancing safety of structured, progressive protocols for improving walking after stroke.

## Methods

### Study design

This cross-sectional study received approval from the Ethics and Research Committee of Santa Casa de Misericórdia Hospital in Porto Alegre (CAAE 64819617.0.0000.5335) and adhered to the Strengthening the Reporting of Observational Studies in Epidemiology (STROBE) checklist.^14^ All participants, deemed competent for decision-making based on their Mini-Mental State Examination (MMSE) scores, provided informed consent before participation.

### Participants and procedures

Participants were sourced from the Santa Casa de Misericórdia de Porto Alegre Hospital—Neurology Service, in Porto Alegre, utilizing both their database and social networks, and then screened against specific eligibility criteria. Inclusion criteria encompassed individuals aged 20 to 80 years with confirmed ischemic or hemorrhagic chronic stroke, identified through brain imaging exams (tomography or magnetic resonance) conducted at least 6 months prior to recruitment. Eligible participants were those with mild (29–34/34), moderate (20–28/34), or severe (0–19/34) hemiparesis, as determined by the Fugl-Meyer score within the lower limb subdivision.^15^ Patients had to have minimal cognitive ability on the MMSE [>20 points (illiterate) or >24 (literates)],^16^ ability to stand up and walk for at least 10 m (with or without walking devices), and no recent episode of fall (at least 3 months before the study engagement). Furthermore, we also excluded individuals with lower limb musculoskeletal disorders that could interfere in gait and significant visual impairment. The control group consisted of 51 healthy individuals, 32 of these individuals also engaged in a steady-state walking task (previously published in Garcia et al.^8^). The control group was recruited following the same procedure indicated for stroke participants. The healthy individuals were matched for age and sex. Exclusion criteria for the control group included a history of neurological or musculoskeletal conditions that caused noticeable gait abnormalities. All participants provided informed consent after being briefed on the study's objectives and potential risks.

This study was carried out at the Movement Analysis and Rehabilitation Laboratory of the Federal University of Health Sciences of Porto Alegre (UFCSPA). Each participant underwent a comprehensive evaluation session that involved collecting personal data (age, hemiparesis side, and time since stroke), physical measurements (weight, height, and BMI), and clinical assessments), all administered by the same researcher.

**Figure 1.**
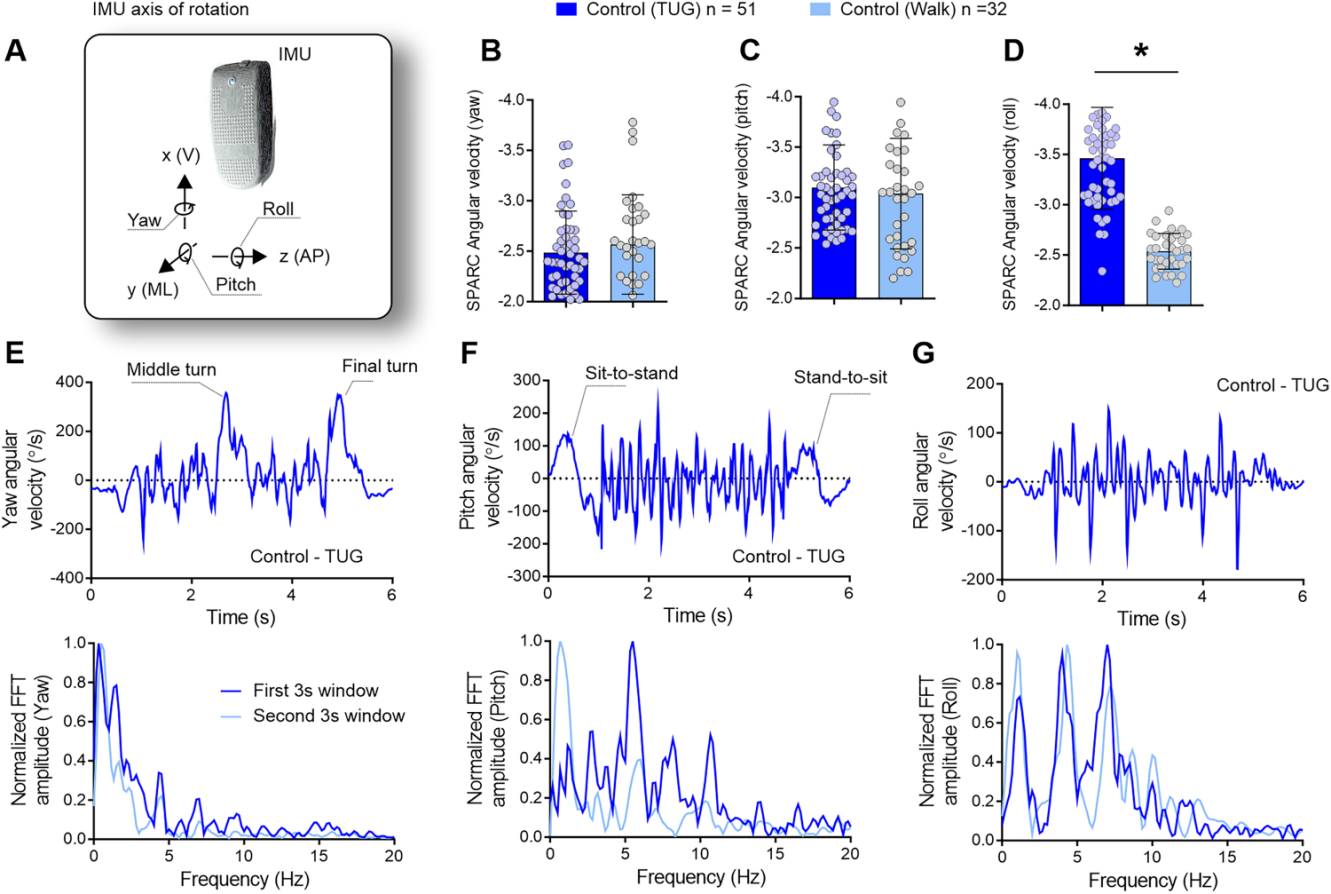
The timed up-and-go test, characterized by sharp turns, sit-to-stand, and stand-to-sit transitions, necessitates intermittent movements in contrast to the smooth and continuous gait observed in steady-state walking. Control participants undertaking the timed-up-and-go test (*n* = 51) were compared to control participants engaged in steady-state walking (*n* = 32). (a) To assess movement smoothness, angular velocities around the yaw, pitch, and roll axes of rotation were used to calculate the spectral arc length. (b–d) While the smoothness of yaw and pitch movements remained similar between the timed-up-and-go test and steady-state walking, movements around the roll axis displayed reduced smoothness during the timed-up-and-go task. (e) During the timed-up-and-go test, yaw angular velocity variations indicate the turning phases (upper panel), with a prevalence of lower-frequency components (lower panel). (f) Pitch angular velocity reflects variations in trunk inclination during sit-to-stand and stand-to-sit transitions (upper panel), encompassing both low and high frequencies (lower panel). (g) Roll angular velocity denotes mediolateral sway necessary for balance maintenance during the timed-up-and-go task, comprising both low and high-frequency components, exceeding those observed during steady-state walking. The spectral distribution reveals that movement smoothness exhibits peaks at both lower and higher frequencies (up to 8 Hz), indicating increased intermittency in movement, as reflected in spectral arc length outcomes in (d). IMU = inertial measurement unit; V = vertical; ML = mediolateral; AP = anteroposterior; FFT = fast Fourier transform; Hz = Hertz; s = seconds. *p < 0.05.

For the assessment of functional mobility, a single miniaturized inertial sensor (G-Sensor^®^, BTS Bioengineering, Italy) was used. The timed-up-and-go test was employed to evaluate functional mobility, wherein participants were instructed to walk safely, without running, and turn toward their affected side. Each participant completed this trial three times, and the average of three trials was used for analysis purposes. The timed-up-and-go test was evaluated using an IMU attached to the waist (L1-L2 spinal segment), and the three directions of angular velocities were utilized for the subsequent spectral arc length analysis (Figure 1(a)). We conducted a comparative analysis of the spectral arc length derived from the angular velocities during the timed-up-and-go test and the 10 m walking test in a subsample of the healthy participants who underwent both assessments. The steady-state walking task was performed as described previously.^8^

Throughout each trial, the sensor recorded accelerations at a sampling rate of 100 Hz, transmitting data via Bluetooth to a PC. Subsequently, a custom LabVIEW routine was used to process the collected data for further analysis.

The spectral arc length analysis was performed using IMU data collected during the standard timed-up-and-go test.^3^ The IMU was attached to the waist, similar to our previous study (for detailed methods, see Garcia et al.^8^ and Supplemental material).

Clinical assessments were performed: Fugl-Meyer Lower Extremity (FM-LE), Modified Ashworth Scale (MAS), and the Activities-specific Balance Confidence (ABC) scale (described in detail in the Supplemental material).

### Statistical analysis

The sample size of 107 participants, comprising 56 chronic stroke survivors and 51 age-matched healthy volunteers, was determined based on prior studies utilizing spectral arc length metrics within our research team. Normality assessment was conducted using Shapiro-Wilk tests (Supplementary Tables 1 and 2), and the choice between parametric and non-parametric analysis was determined accordingly. One-way ANOVA with Tukey’s post hoc analysis was employed to compare spectral arc length mean differences between the stroke groups (categorized as mild/moderate or severe motor impairment) and the control group (roll component), which was deemed robust enough to handle deviations from normality observed in the yaw and pitch components. The Kruskal-Wallis test with Dunn's multiple comparisons test (corrected for multiple comparison using statistical hypothesis testing) was utilized to compare the mean differences in timed-up-and-go test duration between the stroke groups (mild/moderate and severe motor impairment) and the control group. Spearman's correlation was utilized to examine the relationships between clinical scores, demographics, and spectral arc length outcomes. We interpreted the strength of correlation as follows: 0.26–0.49 = weak; 0.5–0.69 = moderate; 0.7–0.89 = strong; and 0.9–1.0 = very strong.^17^ Data were presented as mean and standard deviation of the mean (SD), with statistical significance set at α < 0.05. All analyses were conducted using SPSS statistical software version 21.0.

## Results

Fifty-six stroke participants and 51 healthy controls were included. Demographic data and clinical characteristics of participants are presented in Supplementary Table 3.

We conducted an analysis of spectral arc length derived from angular velocities during the timed-up-and-go test (*n* = 51) and compared to data from a subsample of the same participants (*n* = 32) who also performed a 10 m walking test.^8^

As anticipated, the timed-up-and-go test, characterized by sharp turns, sit-to-stand, and stand-to-sit transitions, exhibited less smooth movements compared to steady-state walking, as reflected in the spectral arc length analysis of the roll angular velocities [Figure 1(d); unpaired *t* test, *n* = 51 (timed-up-and-go: present study) and *n* = 32 (retrospective data); *t* = 10.10, df = 83; *p* < 0.0001]. There were no differences in the yaw [Figure 1(b); unpaired *t* test, *n* = 51 (timed-up-and-go: present study) and *n* = 32 (retrospective data); *t* = 0.805, df = 83; *p* = 0.423] and pitch [Figure 1(c); unpaired *t* test, *n* = 51 (timed-up-and-go: present study) and *n* = 32 (retrospective data); *t* = 0.575, df = 83; *p* = 0.567]. This finding indicates increased movement intermittency, specifically in the mediolateral roll of the body during the timed-up-and-go test compared to steady-state walking.

Representative graphs illustrating a control participant with the lowest spectral arc length values are depicted in Figure 1(e)–(g). The yaw angular velocity, depicting the midway and final turns of the timed-up-and-go test (Figure 1(e), upper panel), exhibited lower frequencies (Figure 1(e), lower panel). The pitch angle, influenced by trunk flexion/extension during sit-to-stand and stand-to-sit movements, as well as the pendular motion during walking, displayed higher frequencies with low complexity (Figure 1(f), upper panel). The roll angular velocity, reflecting the mediolateral sway of the body during step-to-step transitions to maintain gait balance in the timed-up-and-go test, exhibited a complex frequency spectrum, including frequencies above 5 Hz—uncommon in steady-state walking (Figure 1(g)).

**Figure 2.**
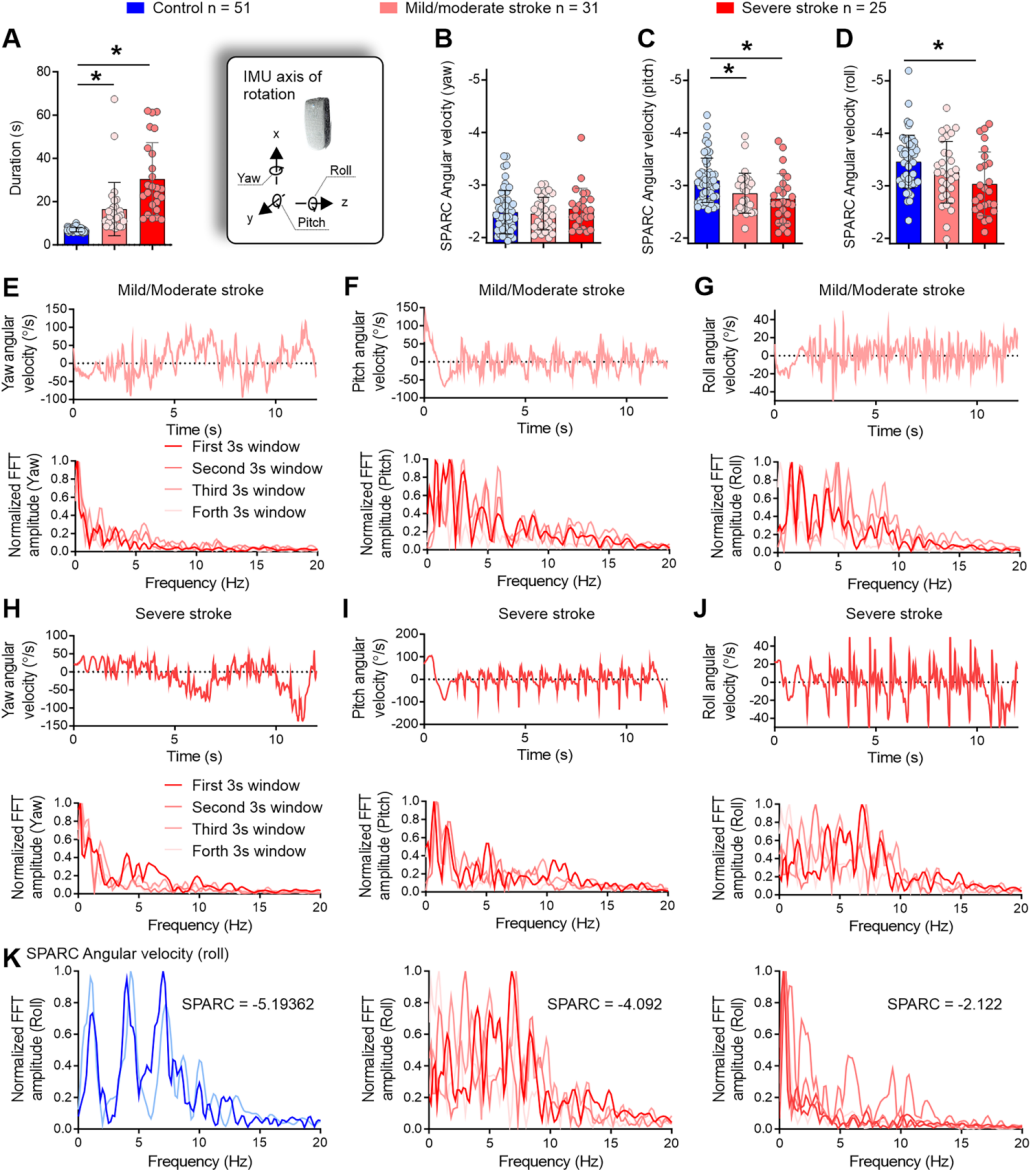
Stroke participants performed the timed-up-and-go test over a longer period of time to maintain smooth mobility, compared to controls. (a) Both mild/moderate and severe stroke individuals took more time to perform the timed-up-and-go test. (b–d) Mild/moderate and severe stroke individuals also displayed smoother movements around the pitch axis (c) and severe stroke participants in the roll axis (d). (e) Mild/moderate individuals performed the middle turn using multiple steps (upper panel) with the prevalence of lower frequencies in the spectral profile (lower panel). (f) In the pitch profile, a reduced trunk angular movement during the stand-to-sit phase was evident (upper panel) alongside the use of intermediate frequencies (≈5 Hz) but with lower/controlled magnitude compared to controls (Figures 1(f) and 2(f), lower panels). (g, upper panel) Low amplitude angular accelerations around the roll axis were evident, compared to controls (Figures 1(g) and 2(g), upper panels). (g, lower panel) Multiple frequency peaks indicated variable intermittency during the timed-up-and-go test for mild/moderate stroke individuals, but the frequencies were kept at a low amplitude compared to controls (Figures 1(g) and 2(g), lower panels). (h–j) Severe stroke participants displayed similar features to mild/moderate stroke individuals. IMU = inertial measurement unit; SPARC = spectral arc length; V = vertical; ML = mediolateral; AP = anteroposterior; FFT = fast Fourier transform; Hz = Hertz; s = seconds. *p < 0.05. (k) Frequency profile differences between a control participant (left) and individuals with stroke (middle and right panels). The control participant (same as Figure 1G) demonstrates high intermittency in roll movements, with a rich frequency profile showing peaks around 2 Hz, 4.5 Hz, and 7.5 Hz, resulting in a spectral arc length of -5.19. In contrast, two cases of stroke are presented: one exhibits significant intermittency (-4.092), while the other shows low intermittency (-2.122).

The Kruskal-Wallis test revealed that stroke participants (both mild/moderate and severe) took significantly longer to complete the timed-up-and-go test compared to healthy controls (Kruskal-Wallis statistic = 80.91, *p* < 0.001; control vs. mild/moderate, mean rank diff. = −44.02, *p* < 0.001; control vs. severe, mean rank diff. = −63.05, *p* < 0.001; Figure 2(a)). Spectral arc length angular velocity around the yaw axis showed no significant difference between stroke participants and healthy controls (Figure 2(b)).

Further analysis using one-way ANOVA confirmed that spectral arc length angular velocity around the pitch and roll axes was higher—less negative—for stroke participants compared to controls [*F*(2, 105) = 6.748, *p* = 0.002; Figure 2(c); *F*(2, 105) = 5.084, *p* = 0.008, Figure 2(d)], indicating smoother mobility in post-stroke individuals. Post hoc tests revealed that both mild/moderate and severe stroke groups exhibited smoother movements around the pitch axis compared to controls (Tukey's multiple comparisons test; mean diff. = −0.2449 and mean diff. = −0.3463, *p* = 0.025 and *p* = 0.002, respectively; Figure 2(c)). This suggests a potentially more cautious approach during sit-to-stand and stand-to-sit movements, as well as during the pendular motion in walking and turning phases. Finally, post hoc tests indicated that the severe stroke group exhibited smoother mobility around the roll axis during the task compared to controls (Tukey's multiple comparisons test; mean diff. = −0.4237, *p* = 0.006; Figure 2(d)), suggesting careful step-to-step transitions to maintain balance.

Illustrative graphs of stroke participants with the lowest spectral arc length values are presented in Figure 2(e)–(j). In the yaw angle, participants with stroke exhibited a distinct pattern, performing the midway turn using multiple steps—central regions of the angular velocity profile during the timed-up-and-go test (Figure 2(e) and (h), upper panels). This can be visualized by the gradual 180-degree shift, which reflects a change in angular velocity during the turning movement, accompanied by several rapid yaw angular velocity shifts that indicate transitions from step to step.

Analysis of the pitch angle indicated reduced trunk movements during the stand-to-sit phase for these participants—initial portion of the angular velocity profile (Figure 2(f) and (i), upper panels). Additionally, the roll angular velocity displayed a lower amplitude compared to controls (approximately 40°/s compared to approximately 150°/s in controls) (Figures 1(g) and 2(g) and (j), upper panels). The spectral distribution highlighted variable intermittency during the timed-up-and-go test for stroke participants with mild/moderate impairment (Figure 2(e)–(j), lower panels). To illustrate the differences between controls and individuals with stroke, Figure 2(k) presents the frequency profile of a control participant (same as Figure 1(g)) who completed the timed-up-and-go test at a fast pace, exhibiting high intermittency in roll movements (characterized by a rich frequency profile with peaks around 2 Hz, 4.5 Hz, and 7.5 Hz; spectral arc length = −5.19). In comparison, two severe stroke cases are shown: one with significant intermittency (spectral arc length = −4.092) and another with low intermittency (spectral arc length = −2.122).

**Figure 3.**
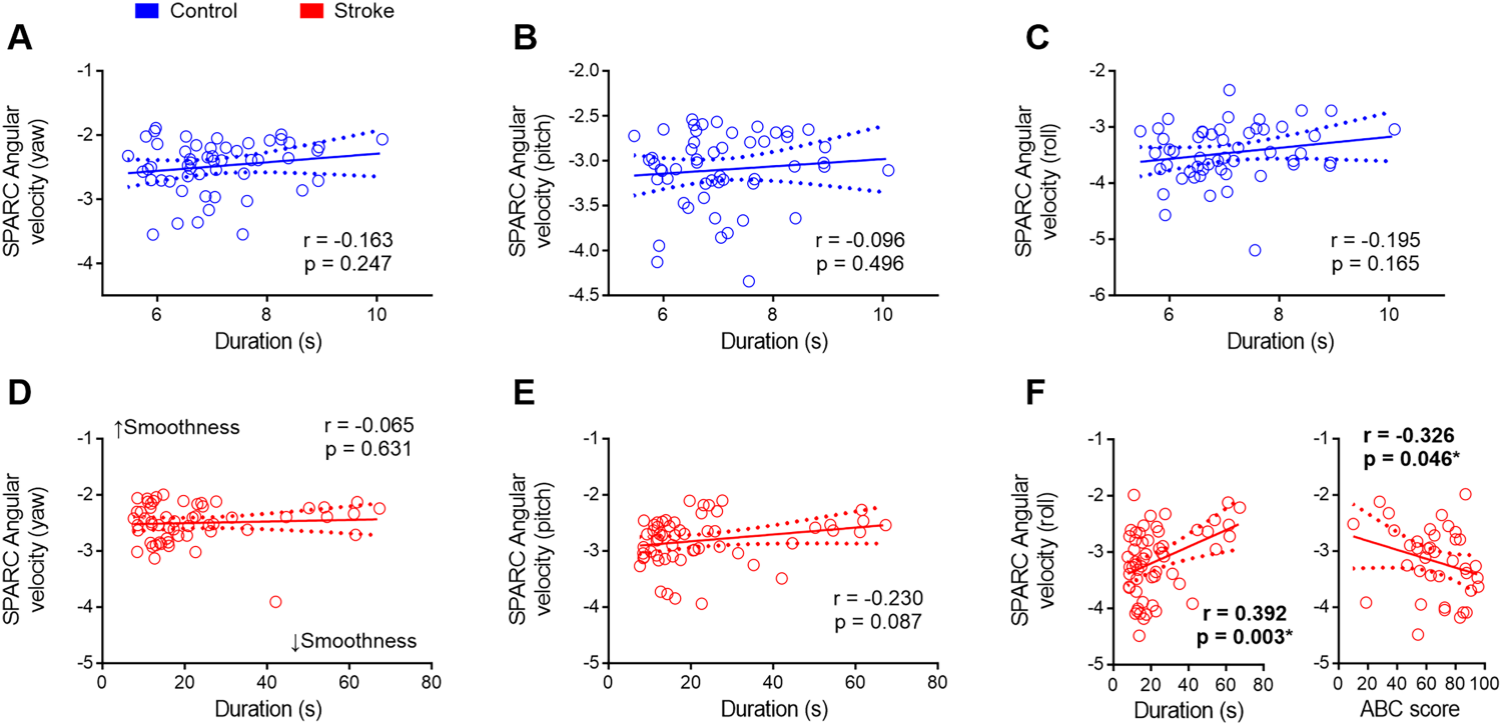
Exploring the relationship between clinical assessments and movement smoothness measurements. (a–c) Movement smoothness remained consistent in control participants regardless of the duration of the task. (d–f) Duration had no discernible impact on smoothness concerning the yaw and pitch axes. However, among stroke individuals, those who invested more time in executing the timed-up-and-go test and those with reduced confidence in balance maintenance exhibited notably smoother movements around the roll axis (f). SPARC = spectral arc length; s = seconds. *p < 0.05.

Supplementary Table 4 and Figure 3 display the correlations between spectral arc length angular velocities and clinical assessments. Spearman correlations unveiled a positive yet weak correlation between timed-up-and-go duration and muscular spasticity, with significant associations observed for plantiflexors (*r* = 0.374; *p* = 0.004), knee extensors (*r* = 0.341; *p* = 0.010), and hip adductors (*r* = 0.362; *p* = 0.006). timed-up-and-go duration also exhibited negative correlations with FM-LE (*r* = −0.605; *p* = 0.001) and ABC score (*r* = −0.696; *p* = 0.001).

Contrastingly, spectral arc length did not show a correlation with FM-LE. However, a positive yet weak correlation surfaced between spectral arc length angular velocity around the pitch axis and hip adductor spasticity (*r* = 0.265; *p* = 0.048), while a negative but weak correlation was noted between spectral arc length angular velocity around the roll axis and ABC score (*r* = −0.326; *p* = 0.046). This suggests that the variations observed in spectral arc length metrics are not attributable to differences in post-stroke impairment severity. Instead, they appear to reflect differences in task demands (as evidenced by data in Figure 1) and the level of confidence in maintaining balance. Specifically, stroke participants who exhibit greater confidence in their balance tend to more actively engage with the demanding aspects of the timed-up-and-go task.

Notably, the MMSE exhibited no correlation with any variable, indicating the absence of cognitive interference in the studied parameters.

To assess the impact of task duration on mobility smoothness metrics, we conducted correlations between spectral arc length and timed-up-and-go duration for healthy control participants. The results indicated that none of the spectral arc length values derived from angular velocities (yaw, pitch, and roll) exhibited a significant correlation with timed-up-and-go duration in control participants (*p* > 0.05, Figure 3(a)–(c)).

Similarly, in the stroke group, there was no significant correlation between spectral arc length angular velocities around the yaw and pitch axes and timed-up-and-go duration (Figure 3(d) and (e)). Conversely, a positive correlation was identified between spectral arc length angular velocity around the roll axis and timed-up-and-go duration in stroke participants (*r* = 0.392; *p* = 0.003, Figure 3(f), left panel). This finding suggests that individuals post-stroke prolong the timed-up-and-go test duration to sustain movement smoothness during the activity.

Interestingly, stroke participants who reported higher confidence in maintaining balance displayed less smooth movements around the roll axis during the timed-up-and-go test (Figure 3(f), right panel). This suggests that those with low confidence in maintaining balance tend to spend more time performing the timed-up-and-go test, employing smoother movements during the activity.

## Discussion

In recent years, clinical guidelines have increasingly advocated for structured, progressive protocols to enhance walking abilities in individuals recovering from stroke.^18–21^ Advances in technology have enabled the monitoring of exercise intensity and therapy progress, as well as opened opportunities to monitor safety concerns during active engagement in daily activities. Our findings underscore significant differences in mobility smoothness between post-stroke individuals and healthy controls during functional tasks, shedding light on potential applications for enhancing real-world mobility monitoring and intervention strategies. In other words, in real life, while engaging with challenging mobility tasks, stroke survivors can monitor the risk involved and adjust accordingly while receiving feedback from wearable devices.

Individuals who had experienced a stroke were more cautious while executing challenging mobility tasks, compared to healthy individuals. The most striking findings of this study indicate that post-stroke individuals take a longer time to complete the timed-up-and-go task to maintain mobility smoothness, avoiding the challenges of the task. Careful stepping resulted in smoothness around the roll axis for the severe group, indicating less intermittency in the mediolateral sway of the body during the timed-up-and-go test. People with chronic stroke are prone to mediolateral balance loss while walking, possibly due to impaired control of paretic foot placement.^22^ A significant proportion of post-stroke falls occur sideways toward the paretic leg, indicating a disruption in the typical strategy for maintaining mediolateral balance.^23^ Mediolateral walking balance in chronic stroke may be impaired by deficits in adjusting swing leg hip abductor activity, and the ability to stabilize foot placement may be reduced due to impaired control in accurately positioning the paretic swing foot.^24,25^ Reduced hip abduction in the paretic leg is linked to wider steps,^26^ likely as a strategy to avoid balance loss. The results of the present study suggest that chronic post-stroke individuals may take longer to complete the task in order to ensure smooth mediolateral adjustments during walking and transitions between the phases of the timed-up-and-go test. Failure to make these adjustments efficiently may contribute to sideways falls toward the paretic leg due to foot misplacement during step-to-step transitions.^23^

These findings reveal the predictive potential of spectral arc length in the roll component for real-time monitoring of mobility during walking and daily activities. In contrast to steady-state walking, individuals post-stroke exhibited spectral arc length values of approximately −3.25 for both walking and the timed-up-and-go test. This stands in contrast to the control group, which demonstrated spectral arc length values of about −2.5 for walking and approximately −3.5 for the timed-up-and-go test. Our findings reveal that post-stroke individuals demonstrating greater confidence in maintaining balance during movement tend to actively participate in challenging tasks like the timed-up-and-go test. Variations in spectral arc length metrics do not correlate with post-stroke impairment severity but rather with task demands and participants’ confidence in balance, especially in engaging with challenging aspects of the task—further supporting its use to quantify safety during mobility tasks.

Similar to other studies,^27^ participants were instructed to walk as fast as possible while prioritizing safety. Despite this directive, severely impaired individuals took ≈30 s to complete the task, a contrast to the ≈7 s taken by their healthy counterparts. This noteworthy difference aligns with our findings on mobility smoothness, suggesting that healthy participants opt for a swifter timed-up-and-go test performance at the expense of lower smoothness in mobility. Notably, smoothness is a hallmark of adept, healthy movement, intricately tied to extensive sensory feedback, and the corresponding fine-tuning of feedforward activation, as seen in activities like steady-state running.^28^ Activities involving abrupt turns and directional changes, akin to those encountered in a soccer match,^29^ showcase the remarkable adaptability of the healthy neuromuscular system to manage movement intermittency. In the current study, stroke participants strategically adopted a more cautious approach, prioritizing mobility smoothness over speed, particularly evident in tasks requiring changes in direction and intermittent movements like the timed-up-and-go test. Compared to other studies this was also in contrast, for example, a study assessing a large cohort of post-stroke individuals (*n* = 91) indicated that the time to complete the timed-up-and-go test was 17 s (first-week post-stroke), 14.5 s (3-month post-stroke), 14.2 s (6-month post-stroke), and 14.7 s (12-month post-stroke).^30^

Finally, the potential of mobility smoothness to predict challenging mobility situations in real-world situations instigates additional investigations into its practicality in real-time, real-world scenarios. Exploring the application of automated detection systems could potentially empower post-stroke individuals, providing timely warnings during daily mobility activities and fostering a safer engagement by alerting users to instances of diminished mobility smoothness.^31^

Translating these findings into clinical practice requires overcoming several challenges to ensure the technology is both usable and meaningful for stroke survivors and clinicians.^32,33^ First, simplifying the integration of wearable technology into routine rehabilitation workflows is crucial—wearable sensors can generate complex data that must be pre-processed.^33^ This also involves designing user-friendly devices that provide clear, real-time feedback on mobility smoothness in a format easily understood by both patients and clinicians.^34^ For stroke survivors, wearable devices should be lightweight, comfortable, and non-intrusive, offering intuitive visual or auditory cues that indicate when mobility smoothness is compromised. Clinicians would benefit from accessible, easy-to-interpret data dashboards that summarize key metrics and trends, allowing them to make informed decisions on patient care in real time. Additionally, incorporating machine learning algorithms could help personalize rehabilitation strategies by identifying individual movement patterns and predicting safety risks based on each patient's unique needs.^33,35^ To bridge this gap, further studies focused on the long-term usability of these devices in clinical settings and their effectiveness in diverse patient populations will be essential.

A few limitations of the present study include the absence of a quantitative assessment of balance and kinematic analysis. We used a phone interview to administer the ABC questionnaire after the study's completion; however, not all participants responded to the interview (*n* = 38). Kinematic assessments of the movements involved in the timed-up-and-go test, along with their relationship to smoothness changes measured by the IMU, would offer a deeper understanding of how movement asymmetries contribute to changes in smoothness during the task.

In conclusion, the findings highlight distinct patterns in movement smoothness across yaw, pitch, and roll axes, underscoring the challenges faced during dynamic tasks like the timed-up-and-go test compared to steady-state walking. Specifically, we observed reduced smoothness in roll movements during the timed-up-and-go test, indicative of increased intermittency necessary for balance maintenance. This study contributes valuable evidence supporting the integration of wearable technology for enhancing clinical assessment and rehabilitation strategies tailored to individual patient needs.

Our findings emphasize the potential of wearable technology to enhance clinical assessment and rehabilitation by providing real-time feedback on movement smoothness. This advancement allows for more tailored and precise rehabilitation strategies, improving safety and helping stroke survivors navigate challenging tasks with greater confidence.

**Clinical messages**

- Stroke survivors use different strategies during dynamic tasks, highlighting difficulties in balance and complex movements.
- Wearable technology effectively measures challenges in dynamic tasks, enabling more precise and safer rehabilitation for stroke survivors.
- Detection systems can offer real-time alerts during mobility rehabilitation, improving safety and helping stroke survivors manage challenging tasks with greater confidence.

## Supplemental Material

sj-docx-1-cre-10.1177_02692155241309083 - Supplemental material for Enhancing safety monitoring in post-stroke rehabilitation through wearable technologies Supplemental material, sj-docx-1-cre-10.1177_02692155241309083 for Enhancing safety monitoring in post-stroke rehabilitation through wearable technologies by Kátia Rech, Maira Jaqueline da Cunha and Ana Paula Salazar, Rosicler da Rosa Almeida, Clarissa Pedrini Schuch, Gustavo Balbinot in Clinical Rehabilitation
