# Supplementary material for: Enhancing safety monitoring in post-stroke rehabilitation through wearable technologies
Source: Clin Rehabil. 2025 Jan 7;39(3):388–98. doi: 10.1177/02692155241309083 (PMC11927009; doi:10.1177/02692155241309083)
Supplement: sj-docx-1-cre-10.1177_02692155241309083 - Supplemental material for Enhancing safety monitoring in post-stroke rehabilitation through wearable technologies [file sj-docx-1-cre-10.1177_02692155241309083.docx]

**Title:** Enhancing Safety Monitoring in Post-Stroke Rehabilitation Through Wearable Technologies.

**Supplementary Material**

**Clinical Assessments**

*Fugl-Meyer Lower Extremity (FM-LE).* The assessment of lower limb motor impairment was conducted using the motor function domain of the FM-LE scale (1). Other domains of the scale such as sensation, balance, joint range of motion, and joint pain were excluded. The motor subscale of FM-LE gauges voluntary movement, velocity, coordination, and reflex activity related to the hip, knee, and ankle. Each item is rated on a score of 0 (cannot be performed), 1 (partially performed), or 2 (performed entirely), with a maximum score of 34 points for the lower limbs. Based on their FMA-LE scores, participants were categorized as having a severe (0 to 19), moderate (20–28), or mild (29 points) lower limb impairment (2).

*Modified Ashworth Scale (MAS)*. To measure the resistance to passive movements and indirectly assess spasticity, we employed the MAS (3). This scale comprises six ordinal values, ranging from 0 (no increase in muscle tone) to 4 (significant stiffness) (4). Participants were evaluated while lying supine and were instructed to remain relaxed during the assessment. Spasticity was assessed in the plantar flexors, knee extensors, and hip adductors.

*Activities-specific Balance Confidence (ABC) scale*. To assess balance confidence we employed the ABC scale (5). The Brazilian-Portuguese version of the activities-specific balance confidence (ABC) scale is a 16-item questionnaire with 11-point subscales (6). Each item measures the level of confidence in performing a specific task without losing balance or becoming unsteady by asking participants to assign scores ranging from 0 (no confidence) to 100 (totally confident). The ABC scale total score is obtained by summing the ratings and dividing by 16. The confidence scores are quantified as follows: >80% indicates a high level of physical functioning; 50-80%, a moderate level of physical functioning; and <50%, a low level of physical functioning (7). The ABC scale was applied by telephone interviews by the same assessor (n = 38). Participants also answered three questions about falls: (1) Did they have experienced a fall last year? How many falls? Where did the fall occur?

**Spectral Arc Length (SPARC) analysis**

Offline signal processing and analyses were performed using LabVIEW (version 8.5; National Instruments, Austin, TX, United States). Angular velocity was measured in three axes of rotation (i.e., yaw, pitch, and roll) by the IMU’s gyroscope during the TUG test. Data was calculated over 3 trials for controls and from 1-4 trials for individuals with stroke (depending on the level of impairment). To determine the onset and offset of movement, the researcher was visually guided by the pitch angle which indicated the initial and final trunk angular movement, during the sit-to-stand (start of the TUG test) and stand-to-sit (end of the TUG test), respectively [detailed methods in (8)]. Movement smoothness was estimated using the SPARC metrics, as described elsewhere (9). This metric is thought to capture the presence of movement arrest periods– intervals of time where there is no movement (i.e., where all derivatives of position are zero) (9).

The SPARC metrics was originally developed for point-to-point reach using input data from kinematics, the estimation of SPARC from IMUs is a relatively novel approach that has been optimized in recent studies (8–12). A main advantage of SPARC over other smoothness metrics is that the analysis is conducted in the frequency-domain, thus, there is a reduced influence of movement amplitude and duration. A conservative non-overlapping, moving window size of 3 seconds (300 frames) was used to analyze the data. The 3-second window represents at least 1 full stride cycle in pathological gait. If the last window was less than 3 seconds, this window was zero-padded to 300 frames and included by the algorithm. Mean subtractions were used to remove the direct current (DC) components from raw angular velocities and whenever signal manipulations caused the drifting of the signal. Each cropped window of 300 frames was zero-padded to 600 frames to increase the FFT frequency resolution, and SPARC was calculated. Subsequently, high frequencies not involved in the TUG test were removed by applying the limits of integration (0–10 Hz bandwidth). The upper and lower limits of integration were set at 0 and 10 Hz to encompass higher frequencies present during the TUG test (12). We calculated the SPARC [$\lambda_{S}^{v}\left( \mathbf{V} \right)$] for each 3 seconds window and performed an average of the SPARC window values throughout the TUG trial.

$\lambda_{S}^{v}\left( \mathbf{V} \right)\triangleq-\int_{0}^{\omega_{c}} \sqrt{\left[ \left( \frac{1}{\omega_{c}} \right)^{2}+\left( \frac{d\hat{V}\left( \omega\right)}{d\omega} \right)^{2} \right]}d\omega$ Equation (1)

$\hat{V}(\omega)=\frac{V (\omega)}{V (0)}$; $V (\omega)=\left| \mathcal{F(}\left\| \mathbf{V}\left( t \right) \right\|_{2}) \right|$

*where* $\boldsymbol{V}$ *(t) represents the velocity (angular) of a movement in the time domain,* $\mathcal{F}$*(·) is the Fourier transform operator, ω_c_ is an adaptive cut-off frequency [see* (9) *for details].*

Movement arrest periods increase the complexity of the frequency composition, which is captured by the SPARC analysis. Because of the negative sign in (1), a lower SPARC value indicates less smoothness and is related to the concept of intermittency of the movement and the temporal dispersion of sub movements (increased frequency domain complexity).

**References**

1. Fugl-Meyer AR. Post-stroke hemiplegia assessment of physical properties. Scand J Rehabil Med Suppl. 1980;7:85–93.

2. Daly JJ, Zimbelman J, Roenigk KL, McCabe JP, Rogers JM, Butler K, et al. Recovery of Coordinated Gait: Randomized Controlled Stroke Trial of Functional Electrical Stimulation (FES) Versus No FES, With Weight-Supported Treadmill and Over-Ground Training. Neurorehabil Neural Repair. 2011 Sep;25(7):588–96.

3. Lindsay C, Kouzouna A, Simcox C, Pandyan AD. Pharmacological interventions other than botulinum toxin for spasticity after stroke. Cochrane Database Syst Rev. 2016 Oct 6;10(10):CD010362.

4. Bohannon RW, Smith MB. Interrater reliability of a modified Ashworth scale of muscle spasticity. Phys Ther. 1987 Feb;67(2):206–7.

5. An S, Lee Y, Lee D, Cho KH, Lee G, Park D sik. Discriminative and predictive validity of the short-form activities-specific balance confidence scale for predicting fall of stroke survivors. J Phys Ther Sci. 2017;29(4):716–21.

6. Marques AP, Mendes YC, Taddei U, Pereira CAB, Assumpção A. Brazilian-Portuguese translation and cross cultural adaptation of the activities-specific balance confidence (ABC) scale. Braz J Phys Ther. 2013 Apr;17(2):170–8.

7. Myers AM, Fletcher PC, Myers AH, Sherk W. Discriminative and Evaluative Properties of the Activities-specific Balance Confidence (ABC) Scale. J Gerontol A Biol Sci Med Sci. 1998 Jul 1;53A(4):M287–94.

8. Figueiredo AI, Balbinot G, Brauner F de O, Schiavo A, Baptista RR, Pagnussat A de S, et al. SPARC Metrics Provide Mobility Smoothness Assessment in Oldest-Old With and Without a History of Falls: A Case Control Study. Front Physiol. 2020;11(540).

9. Melendez-Calderon A, Shirota C, Balasubramanian S. Estimating Movement Smoothness From Inertial Measurement Units. Front Bioeng Biotechnol. 2021 Jan 14;8:558771.

10. Beck Y, Herman T, Brozgol M, Giladi N, Mirelman A, Hausdorff JM. SPARC : a new approach to quantifying gait smoothness in patients with Parkinson ’ s disease. J NeuroEngineering Rehabil. 2018;15(49):1–9.

11. Figueiredo AI, Balbinot G, Brauner FO, Schiavo A, de Souza Urbanetto M, Mestriner RG. History of falls alters movement smoothness and time taken to complete a functional mobility task in the oldest-old: A case-control study. Exp Gerontol. 2022 Oct;167:111918.

12. Pinto C, Schuch CP, Balbinot G, Salazar AP, Hennig EM, Kleiner AFR, et al. Movement smoothness during a functional mobility task in subjects with Parkinson’s disease and freezing of gait - An analysis using inertial measurement units. J NeuroEngineering Rehabil. 2019;16(1):1–14.

**Supplementary Table 1**

| **Supplementary table 1.** The Shapiro-Wilk normality test indicated the use of parametric or non-parametric statistics. | | | | | | | | |
| --- | --- | --- | --- | --- | --- | --- | --- | --- |
|  |  |  |  |  |  |  |  |  |
|  |  | SPARC Angular velocity (yaw) | | |  | SPARC Angular velocity (pitch) | | |
|  |  | Control | Mild/Moderate stroke | Severe stroke |  | Control | Mild/Moderate stroke | Severe stroke |
| W |  | 0.922 | 0.947 | 0.854 |  | 0.921 | 0.916 | 0.944 |
| P value |  | **0.002*** | 0.126 | **0.002*** |  | **0.002*** | **0.018*** | 0.180 |
| Passed normality test? |  | **No** | Yes | **No** |  | **No** | **No** | Yes |
|  |  |  |  |  |  |  |  |  |
|  |  |  |  |  |  |  |  |  |
|  |  | SPARC Angular velocity (roll) | | |  | Duration | | |
|  |  | Control | Mild/Moderate stroke | Severe stroke |  | Control | Mild/Moderate stroke | Severe stroke |
| W |  | 0.959 | 0.985 | 0.930 |  | 0.947 | 0.601 | 0.871 |
| P value |  | 0.074 | 0.937 | 0.088 |  | **0.022*** | < 0.001* | **0.004*** |
| Passed normality test? |  | Yes | Yes | Yes |  | **No** | **No** | **No** |
|  |  |  | | |  |  | | |
| *SPARC = Spectral Arc Length; (bold)*p < 0.05* | | | | | | | | |

**Supplementary Table 2**

| **Supplementary table 2.** The Shapiro-Wilk normality test indicated the use of parametric or non-parametric statistics. | | | | | | | | | | |
| --- | --- | --- | --- | --- | --- | --- | --- | --- | --- | --- |
|  |  |  |  |  |  |  |  |  |  |  |
|  |  | Spasticity (MAS) | | |  | FM score |  | MMSE |  | ABC score |
|  |  | Plantiflexors | Knee extensors | Hip adductors |  |  |  |  |  |  |
| W |  | **0.891** | **0.854** | **0.862** |  | **0.955** |  | **0.828** |  | 0.944 |
| P value |  | **<0.001*** | **<0.001*** | **<0.001*** |  | **0.037*** |  | **<0.001*** |  | 0.055 |
| Passed normality test? |  | **No** | **No** | **No** |  | **No** |  | **No** |  | Yes |
|  |  |  |  |  |  |  |  |  |  |  |
| *MAS = Modified Ashworth Scale; FM-LE = Fugl-Meyer Lower Extremity; MMSE = Mini-mental state examination; ABC scale = Activities-specific balance confidence scale; (bold)*p < 0.05* | | | | | | | | | | |

**Supplementary Table 3**

| **Supplementary Table 3.** Demographics (Control group, n = 51; Stroke group, n = 56). | | | | | | | | | |
| --- | --- | --- | --- | --- | --- | --- | --- | --- | --- |
|  |  |  |  |  |  |  |  |  |  |
| **Stroke group (n = 56)** | **FM-LE** | **Gender** | **Age** | **Stroke type** | **Time after stroke (Mo)** | **Affected hemibody** | **Spasticity (plantarflexion)** | **Spasticity (extensors)** | **ABC Score (n=38)** |
| Participant 1 | 11 | Female | 56 | Ischemic | 40 | Left | 4 | 1.5 | 28.12 |
| Participant 2 | 11 | Female | 64 | Ischemic | 64 | Right | 4 | 3 | 55.62 |
| Participant 3 | 12 | Male | 46 | Ischemic | 23 | Right | 0 | 0 |  |
| Participant 4 | 13 | Female | 68 | Ischemic | 39 | Right | 3 | 1.5 | 48.75 |
| Participant 5 | 14 | Male | 54 | Ischemic | 19 | Left | 4 | 3 | 53.75 |
| Participant 6 | 14 | Female | 62 | Ischemic | 24 | Left | 4 | 2 | 83.12 |
| Participant 7 | 14 | Male | 79 | Ischemic | 15 | Left | 0 | 1 |  |
| Participant 8 | 15 | Female | 49 | Ischemic | 24 | Right | 3 | 1 | 87.5 |
| Participant 9 | 15 | Male | 54 | Hemorrhagic | 66 | Right | 3 | 2 | 83.12 |
| Participant 10 | 15 | Female | 57 | Ischemic | 19 | Right | 2 | 1 | 80 |
| Participant 11 | 15 | Female | 60 | Ischemic | 41 | Left | 4 | 1 | 38.12 |
| Participant 12 | 15 | Female | 56 | Ischemic | 18 | Right | 1 | 0 | 90.62 |
| Participant 13 | 15 | Female | 60 | Ischemic | 6 | Left | 4 | 4 | 18.75 |
| Participant 14 | 16 | Male | 62 | Ischemic | 14 | Right | 3 | 3 |  |
| Participant 15 | 16 | Female | 59 | Hemorrhagic | 68 | Right | 4 | 2 | 34.37 |
| Participant 16 | 16 | Female | 68 | Ischemic | 13 | Left | 4 | 2 |  |
| Participant 17 | 16 | Female | 59 | Ischemic | 28 | Right | 1.5 | 0 | 65.62 |
| Participant 18 | 17 | Female | 44 | Hemorrhagic | 21 | Right | 2 | 0 |  |
| Participant 19 | 17 | Female | 54 | Ischemic | 22 | Left | 3 | 1 | 65.62 |
| Participant 20 | 17 | Female | 60 | Ischemic | 84 | Left | 3 | 3 | 70.62 |
| Participant 21 | 17 | Female | 69 | Ischemic | 8 | Left | 3 | 0 |  |
| Participant 22 | 18 | Male | 58 | Hemorrhagic | 14 | Left | 4 | 4 | 75 |
| Participant 23 | 18 | Male | 56 | Ischemic | 56 | Right | 4 | 0 |  |
| Participant 24 | 18 | Male | 50 | Ischemic | 54 | Left | 1 | 0 | 63.12 |
| Participant 25 | 18 | Male | 65 | Ischemic | 26 | Right | 3 | 1 | 10 |
| Participant 26 | 20 | Female | 49 | Hemorrhagic | 45 | Left | 3 | 2 | 56.25 |
| Participant 27 | 20 | Female | 37 | Hemorrhagic | 23 | Right | 3 | 0 | 85.31 |
| Participant 28 | 20 | Female | 44 | Hemorrhagic | 96 | Left | 4 | 3 | 68.75 |
| Participant 29 | 21 | Female | 45 | Ischemic | 80 | Left | 0 | 1 |  |
| Participant 30 | 21 | Female | 49 | Ischemic | 36 | Left | 2 | 1 |  |
| Participant 31 | 21 | Female | 47 | Ischemic | 35 | Left | 1.5 | 1 | 54.37 |
| Participant 32 | 21 | Female | 72 | Ischemic | 36 | Right | 3 | 0 |  |
| Participant 33 | 21 | Female | 47 | Ischemic | 31 | Left | 3 | 1 |  |
| Participant 34 | 21 | Male | 66 | Ischemic | 23 | Right | 1.5 | 0 |  |
| Participant 35 | 22 | Female | 58 | Ischemic | 41 | Right | 4 | 3 | 62.5 |
| Participant 36 | 22 | Male | 38 | Ischemic | 55 | Right | 4 | 1 | 53.75 |
| Participant 37 | 23 | Male | 51 | Hemorrhagic | 9 | Right | 4 | 3 |  |
| Participant 38 | 23 | Female | 72 | Ischemic | 20 | Left | 2 | 1 | 78.12 |
| Participant 39 | 23 | Female | 66 | Ischemic | 96 | Right | 3 | 1.5 | 59.37 |
| Participant 40 | 25 | Female | 74 | Ischemic | 6 | Right | 2 | 1.5 |  |
| Participant 41 | 25 | Female | 75 | Ischemic | 39 | Left | 0 | 0 | 93.75 |
| Participant 42 | 25 | Male | 56 | Ischemic | 17 | Right | 1 | 0 | 46.87 |
| Participant 43 | 25 | Female | 35 | Hemorrhagic | 40 | Left | 4 | 3 | 95 |
| Participant 44 | 26 | Female | 66 | Ischemic | 49 | Left | 1 | 0 | 72.5 |
| Participant 45 | 27 | Female | 58 | Ischemic | 59 | Right | 2 | 1 | 72.5 |
| Participant 46 | 27 | Female | 51 | Ischemic | 67 | Right | 0 | 0 | 95.62 |
| Participant 47 | 27 | Male | 70 | Ischemic | 52 | Left | 1 | 0 | 86.87 |
| Participant 48 | 28 | Female | 63 | Ischemic | 7 | Left | 2 | 1 | 61.25 |
| Participant 49 | 28 | Male | 66 | Ischemic | 39 | Left | 0 | 0 |  |
| Participant 50 | 29 | Male | 34 | Hemorrhagic | 36 | Left | 2 | 1 |  |
| Participant 51 | 29 | Male | 29 | Ischemic | 20 | Left | 2 | 1 |  |
| Participant 52 | 29 | Male | 72 | Ischemic | 31 | Right | 0 | 1 |  |
| Participant 53 | 30 | Male | 69 | Hemorrhagic | 87 | Left | 2 | 0 | 80 |
| Participant 54 | 30 | Male | 53 | Ischemic | 21 | Left | 2 | 1 | 86.87 |
| Participant 55 | 31 | Female | 65 | Hemorrhagic | 22 | Left | 1 | 0 | 87.5 |
| Participant 56 | 32 | Female | 50 | Ischemic | 6 | Left | 1.5 | 1 | 62.5 |
|  |  |  |  |  |  |  |  |  |  |
| **Mean** | #### |  | ### |  | #### |  | 2.38 | 1.20 | 66.09 |
| **SD** | 5.70 |  | ### |  | #### |  | 1.35 | 1.14 | 21.16 |
|  |  |  |  |  |  |  |  |  |  |
| Count |  | 20 (M) / 36 (F) |  | 44 (I) / 12 (H) |  | 25 (R) / 31 (L) |  |  |  |
|  |  |  |  |  |  |  |  |  |  |
|  |  |  |  |  |  |  |  |  |  |
| **Control group (n = 51)** | | |  |  |  |  |  |  |  |
|  |  |  |  |  |  |  |  |  |  |
| Mean | NA |  | ### | NA | NA | NA | NA | NA | NA |
| SD | NA |  | 8.97 | NA | NA | NA | NA | NA | NA |
|  |  |  |  |  |  |  |  |  |  |
| Count |  | 18 (M) / 33 (F) |  |  |  |  |  |  |  |
|  | | | | | | | | | |
| *Notes: FM-LE = Fugl-Meyer lower extremity; Mo = Months; M = Male; F = Female; I = Ischemic; H = hemorrhagic; R = Righ hemobody; L = Left hemobody.* | | | | | | | | | |

**Supplementary Table 4**

| **Supplementary Table 4.** Correlations between movement smoothness and clinical tests for post-stroke individuals (n = 56). | | | | | | | | | | |
| --- | --- | --- | --- | --- | --- | --- | --- | --- | --- | --- |
|  |  | Spasticity (MAS) | | |  | FM-LE |  | MMSE |  | ABC score |
|  |  |  |  |  |  |  |  |  |  |  |
|  |  | Plantar  flexors | Knee Extensors | Hip  Adductors |  |  |  |  |  |  |
| SPARC Angular velocity (yaw) | p | 0.993 | 0.978 | 0.938 |  | 0.139 |  | 0.336 |  | 0.781 |
|  | r | 0.001 | 0.004 | -0.011 |  | 0.200 |  | 0.134 |  | -0.047 |
|  |  |  |  |  |  |  |  |  |  |  |
| SPARC Angular velocity (pitch) | p | 0.267 | 0.251 | **0.048*** |  | 0.629 |  | 0.481 |  | 0.103 |
|  | r | 0.151 | 0.156 | **0.265** |  | -0.066 |  | -0.098 |  | -0.268 |
|  |  |  |  |  |  |  |  |  |  |  |
| SPARC Angular velocity (roll) | p | 0.266 | 0.487 | 0.055 |  | 0.850 |  | 0.412 |  | **0.046*** |
|  | r | 0.151 | 0.095 | 0.258 |  | -0.026 |  | -0.114 |  | **-0.326** |
|  |  |  |  |  |  |  |  |  |  |  |
| Timed-up-and-go duration (seconds) | p | **0.004*** | **0.010*** | **0.006*** |  | **<** **0.001*** |  | 0.051 |  | **< 0.001*** |
|  | r | **0.374** | **0.341** | **0.362** |  | **-0.605** |  | -0.267 |  | **-0.696** |
|  |  |  |  |  |  |  |  |  |  |  |
| *MAS =* *Modified Ashworth Scale; FM-LE =* *Fugl-Meyer Lower Extremity; MMSE = Mini-mental state examination; ABC = Activities-specific balance confidence scale; Spearman correlation; (bold)*p < 0.05* | | | | | | | | | | |
